# Iron Deficiency Anemia and Its Impact on Oral Health—A Literature Review

**DOI:** 10.3390/dj12060176

**Published:** 2024-06-07

**Authors:** Kabilan Velliyagounder, Krupa Chavan, Kenneth Markowitz

**Affiliations:** Department of Oral Biology, Rutgers School of Dental Medicine, 110 Bergen Street, Newark, NJ 07103, USA; kpc101@gsbs.rutgers.edu (K.C.); markowkj@sdm.rutgers.edu (K.M.)

**Keywords:** iron deficiency, iron deficiency anemia, oral diseases, oral manifestations, periodontal diseases, dental caries, oral candidiasis

## Abstract

Oral disease interventions primarily focus on behavioral changes like dietary improvements and ensuring better oral hygiene. However, recognizing the influence of biological factors, including genetics and early-life nutrition, is crucial. Iron deficiency (ID) and its advanced form, iron deficiency anemia (IDA), affect nearly two billion people globally, especially children and pregnant women. We conducted a comprehensive search using Medline via EndNote and Web of Science, employing keywords related to iron deficiency anemia (IDA), and we identified 36 studies deemed relevant for inclusion in this literature review. IDA prevalence is notably high among pregnant women and young children. Both IDA and early-childhood caries (ECC) disproportionately affect impoverished populations, highlighting the socioeconomic dimension of this issue. IDA presents with various oral mucosal changes and is closely linked to candidiasis. Additionally, IDA can hinder tooth development and weaken the immune response. Multiple population surveys have revealed a significant association between ECC and IDA. While some studies have explored the IDA–periodontal disease link, the current evidence is relatively limited in its robustness. In conclusion, more comprehensive longitudinal studies are essential to deepen our understanding of the IDA–oral disease connection. Investigating the underlying biological mechanisms is critical to developing effective interventions, particularly for vulnerable populations affected by IDA.

## 1. Introduction

Iron deficiency (ID) is the most prevalent nutritional deficiency worldwide, accounting for approximately 50% of all cases of anemia. Iron deficiency anemia (IDA) is a pervasive health concern, affecting a staggering two billion individuals globally, with a significant majority residing in non-industrialized countries [1]. The prevalence of IDA exhibits variations across races, genders, and age groups, thereby presenting a complex epidemiological picture. In the United States, statistics reveal notable disparities in IDA rates. Among adults, approximately 2% of men, up to 12% of white women, and around 20% of black women grapple with this condition. Mexican American women face a strikingly higher prevalence, with a rate 2.3 times greater than that of non-Hispanic Caucasian women [2]. Alarming trends emerge in pediatric populations as well, where 9% of children aged 12–36 months experience ID, and 33% of them are at risk of developing anemia [1]. The detrimental consequences of IDA encompass heightened fatigue and reduced work capacity, underscoring the profound impact of this deficiency on daily life and productivity. Prenatal IDA can result in increased fetal morbidity and mortality as well as hindered fetal growth [3]. Episodic occurrences of IDA can manifest during episodes of blood loss, such as during heavy menstruation or blood donation [4]. Additionally, IDA can afflict individuals with conditions like gastric ulcers, gastrointestinal carcinomas, and those who use aspirin or non-steroidal anti-inflammatory drugs (NSAIDs). Reduced iron absorption may contribute to IDA in patients with gastrectomy, celiac disease, vitamin A or B12 deficiency, and chronic infections like Helicobacter pylori infection, tuberculosis, or HIV [5].

**Figure 1:**
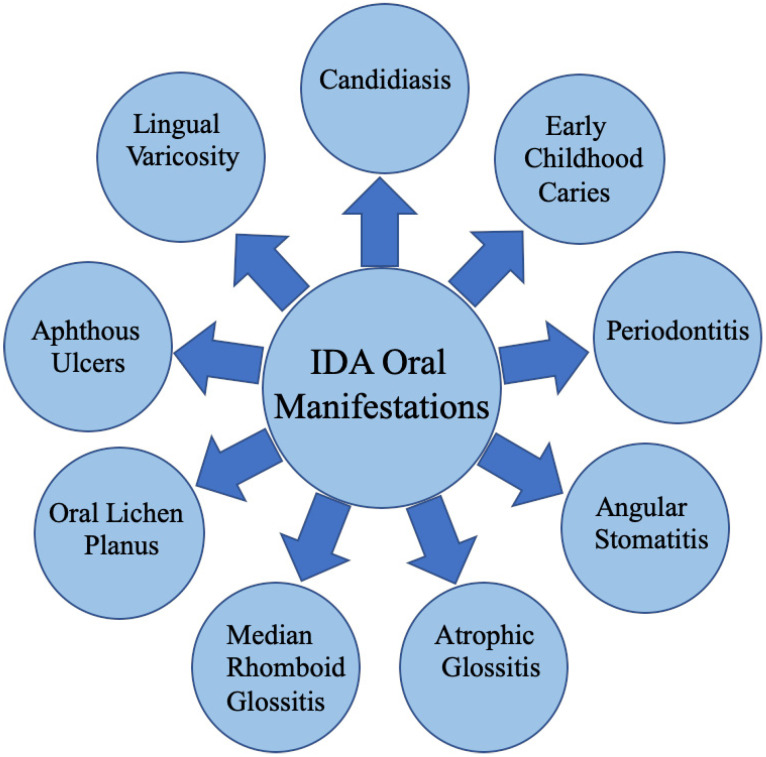
The scope of oral diseases associated with IDA.

The World Health Organization recognizes a spectrum of iron statuses in individuals, ranging from IDA to iron overload, with various stages of ID and normal iron levels in between [1]. In the realm of healthcare, the early detection of oral manifestations during oral cavity examinations has proven invaluable for diagnosing underlying systemic conditions. These oral manifestations may precede or follow findings from other bodily sites, thereby facilitating early diagnosis and timely treatment [6]. While many oral disorders stem from plaque-related factors, the oral mucosa can undergo alterations as a consequence of systemic diseases, often serving as the first site to exhibit signs of such conditions. It is imperative for primary care physicians and dentists to be well versed in the oral manifestations of systemic diseases, enabling them to recognize and appropriately manage these signs and symptoms. A comprehensive oral examination should encompass the assessment of mucosal changes, including pallor and pigmentation; the scrutiny of the mucosal surface and palate for lesions; the evaluation of periodontal health, with attention paid to inflammation and bleeding; and an examination of the tongue, gingiva, and overall dental condition. Oral findings indicative of IDA may encompass severe dental caries, chronic periodontitis, candidiasis, burning sensations and redness of the tongue, mucosal pallor, atrophy of the lingual papillae, atrophic glossitis, dysphagia, and an elevated risk of developing oral cancer (Figure 1). 

## 2. Methodology

We conducted a comprehensive literature search to investigate the association between iron deficiency anemia (IDA) and oral health issues, utilizing EndNote 21.2 to manage records from Medline and Web of Science. The search targeted studies published up to 3 May 2024, focusing on cross-sectional, case–control, and cohort studies related to dental caries, periodontitis, oral thrush, and IDA. The medical subject headings (MeSH) used included the following: “child”, “children”, “dental caries”, “periodontitis”, “*Candida albicans*”, “iron deficiency”, “iron deficiency anemia”, “IDA”, “ID”, “ECC and IDA”, “periodontitis and IDA”, “Candida albicans and IDA”, “ECC or IDA”, “periodontitis or IDA”, “*Candida albicans* or IDA”.

Selection Criteria: The primary research question addressed whether IDA is associated with ECC, Candida albicans infections, or periodontitis. The PECO framework was defined as follows: There are two possible PECOs, one based on dental disease being a risk factor for IDA and a second one based on IDA being a risk factor for dental disease. The second one is simpler.

Populations:

1. Children with ECC;
2. Adults with periodontal disease;
3. Adults and children with Candida albicans infections;
4. Adults and children with oral ulcerations.

Exposure: Presence of ECC, periodontitis, oral thrush, oral candidiasis, or other oral manifestations.

Comparison: Absence of these conditions.

Outcome: IDA, assessed by hemoglobin (Hb) levels, serum ferritin, and mean corpuscular volume (MCV).

The PECO could be as follows:

Population: Adults or children at risk for ECC, periodontitis, candidiasis, or oral ulcerations.

Exposure: Diets containing inadequate levels of iron resulting in iron deficiency or IDA as determined by hematological examination.

Control: Diets containing adequate iron levels.

Outcomes:

1. Prevalence of ECC;
2. Prevalence of periodontal disease;
3. Prevalence of candidiasis;
4. Prevalence of oral ulcerations.

Exclusion Criteria: Excluded materials included books, policy briefs, reviews, theses, dissertations, and non-peer-reviewed articles.

**Figure 2:**
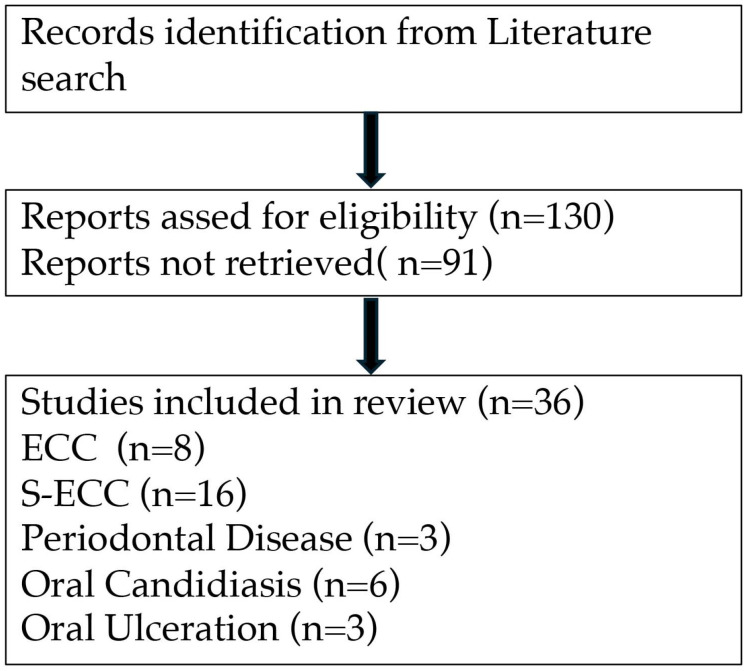
Identification of studies vis databases.

Selection of Studies: All titles and abstracts were independently assessed against the inclusion criteria by the review authors. Discrepancies were resolved by consensus. This process identified 130 relevant articles, with 36 studies selected for detailed review after a selective screening process. These included 36 human studies, encompassing case–control and cross-sectional designs, and 6 animal studies. The breakdown of studies included 3 on periodontal disease, 24 on dental caries (compared against 6 animal studies), 6 on oral candidiasis, and 3 on ulcerations. This review aims to elucidate the connections between IDA and specific oral health conditions, enhancing our understanding of their interrelations and potential implications for clinical practice and public health strategies (Figure 2). Detailed study inclusion and exclusion process is presented in Figure 2.

## 3. Oral Manifestations of IDA and Association of IDA with ECC and IDA

Dental caries, characterized by the degradation of tooth enamel and dentin due to bacterial acids produced during the metabolism of sugars and other carbohydrates, is a complex multifactorial disease. Its development begins with an ecological imbalance in the bacterial biofilm of the oral cavity, favoring acid-producing and acid-tolerant organisms. Factors such as adherence to a high-sugar diet, poor oral hygiene, reduced saliva flow, and the absence of immunological protective factors can contribute to the shift of the biofilm toward a cariogenic flora. Within this context, Streptococcus mutans, a Gram-positive facultative anaerobic bacterium belonging to the viridans group of oral streptococci, stands out as a major contributor to the bacterial flora associated with dental caries [7].

While the role of diet in caries development is well established, the influence of nutrition on caries risk, particularly concerning the role of ID, is not as thoroughly understood. Nutrition can affect tooth development and the host’s defenses against harmful bacteria, thereby influencing the microbial colonization of teeth and their resistance to cariogenic organisms [8]. Several studies have explored the relationship between malnutrition, including ID, and the etiology of caries. Notably, both caries and malnutrition disproportionately affect economically disadvantaged populations. However, the specific role of ID in caries etiology and its potential contribution to the development of severe dental decay leading to IDA remain less clear. In the pediatric population, particularly among preschool-aged children, ECC and S-ECC are prevalent issues, affecting approximately 4.5 million children annually [9]. ECC is defined by the American Academy of Pediatric Dentistry as the presence of one or more decayed, missing (due to caries), or filled tooth surfaces in any primary tooth in a child under the age of six. S-ECC, on the other hand, specifically refers to the presence of smooth-surface caries in a child younger than three years of age [10]. Data from the National Health and Nutrition Examination Survey revealed that in 1999–2004 surveys, the prevalence of caries in children aged 2 to 5 years was 28%, with a significant portion of these cases (72%) being untreated [11].

Children affected by ECC often experience malnutrition, with their weight typically falling to 80% or less of their ideal weight. They may also suffer from anemia and exhibit altered patterns of somatic growth [12]. It is noteworthy that Kazal reported a lower iron intake among toddlers aged 1–3 compared to other age groups, indicating a potential overlap between IDA and ECC in this population [13]. The relationship between caries, diet, and nutrition is intricately linked, with poor nutrition serving as both a cause and consequence of dental caries. Many studies have explored the association between iron deficiency and ECC. Abdallah et al. conducted a cross-sectional study involving 160 preschool children and observed a correlation between their Hb levels and decayed, missing, and filled tooth (dmft) index scores. The anemic children in this study exhibited significantly higher mean dmft index scores when compared to their non-anemic counterparts [14].

In contrast, Nur et al. evaluated 160 preschool children with S-ECC undergoing full dental rehabilitation. Their findings did not reveal a significant association between Hb or HCT levels and S-ECC. However, they did observe a significantly lower MCV in S-ECC patients, suggesting potential variations in red blood cell size [15]. Additional studies have also indicated a strong relationship between ID and caries among children. Clarke et al. conducted research focused on the nutritional status of children with S-ECC. They examined 56 children between the ages of 2 and 6 who had S-ECC and found that approximately 74% of these children had blood Hb levels ranging from low to borderline and that 80% exhibited low serum ferritin levels [16]. These findings collectively suggest a notable connection between iron deficiency and dental caries in the pediatric population. Sadeghi et al. conducted a double-blind randomized cross-sectional study on 204 preschool children to investigate the association between ECC and IDA. They observed an inverse relation between the serum iron level and the dmft index score in preschool children [17]. Koppal et al. conducted a cross-sectional study to investigate the relationship between IDA and S-ECC. They concluded that ECC and IDA are certainly correlated, but a longitudinal study is recommended to further investigate the role of IDA as a risk factor for ECC [18]. Tang et al. examined 101 children to understand the relation between IDA and S-ECC. A multivariable logistic regression analysis found that children with dmfs that are higher or equal to 35 have a 7.25-fold higher risk of developing IDA [19]. Jayakumar and Gurunathan et al. examined the relation between the serum ferritin level of children under the age of six and the severity of ECC. They included 79 children with ECC and 35 caries-free children as the control group and found significantly lower mean ferritin levels in the ECC patients when compared to the caries-free group [20]. Venkatesh Babu et al. evaluated the relation between serum iron and ferritin levels and dental caries in children between ages 3 and 12 [21]. They concluded that there is a significant association between the dmft index and serum iron levels but not ferritin levels. In addition, Bansal et al. conducted another case–control study and found a strong association between IDA and S-ECC [22]. Mohamed et al. conducted a cross-sectional study involving children diagnosed with IDA and showed a positive correlation with caries incidence [23]. It has been reported that children who received 3 months of IDA treatment demonstrated an improvement in their salivary pH level and its buffering capacity [24]. Schroth et al. recruited a total of 266 children under the age of six for their study. They found that children with S-ECC had significantly lower levels of Hb and ferritin and were over six times more likely to have IDA when compared to their peers who did not have dental caries. The frequency of IDA was significantly more prevalent in the S-ECC children in comparison to the caries-free control group [10]. Atri et al. investigated potential correlations between S-ECC and serum iron and ferritin levels in children. From a pool of 688 children, 82 were selected based on their decayed, missing, and filled primary teeth (dmft) scores. The results indicated that in the S-ECC group, Hb, serum iron, and serum ferritin levels; total iron-binding capacity (TIBC); and unsaturated iron-binding capacity (UIBC) were lower compared to the control group, but these differences did not reach statistical significance. However, statistically significant differences were observed only in the dmft and UIBC values. These findings suggest a potential inverse relationship between S-ECC and serum iron and serum ferritin levels, although further research is needed to establish conclusive evidence [25].

Shaoul et al. published a short communication suggesting that S-ECC is a risk factor for IDA. They proposed that effective dental treatment can lead to the correction of IDA, indicating a strong association between S-ECC and IDA. Similar results were obtained by Nagarajan et al., who conducted a study on 30 children with S-ECC undergoing full-mouth rehabilitation. Their study involved collecting blood samples before and three months after treatment and comparing the values. The results showed statistically significant improvements in Hb, MCV, MCHC, and ferritin levels and the children’s weight post-treatment. These findings suggest that S-ECC and IDA frequently affect the same individuals, with ECC potentially leading to IDA by impacting a child’s nutritional status [26,27]. Additionally, Acharya et al. observed that untreated dental caries may have systemic health effects. One hypothesis related to children with S-ECC is that they tend to have low Hb levels, which may be attributed to the inflammatory response accompanying severe dental caries, especially cases involving pulpitis or abscesses. This inflammation triggers the production of cytokines, which may inhibit erythropoiesis and reduce Hb levels. Moreover, children with dental pain from S-ECC may have altered eating habits, and a highly cariogenic diet may lack nutritional value, potentially leading to poor growth. Poor sleep quality due to dental pain may also contribute to a decreased production of glucocorticoids, further affecting growth. These observations highlight the potential systemic impact of untreated dental caries, especially in cases of S-ECC [28].

Overall, these studies indicate a correlation between IDA and ECC, but they do not definitively establish whether one condition causes the other. However, it is suggested that iron and other nutritional deficiencies could affect the development of teeth, making them more susceptible to caries. The biological basis of this association is not fully understood, but it is clear that nutritional deficiencies play a role in the relationship between ECC and IDA in children. Mahadevan conducted a study assessing salivary protein levels as an indicator of nutritional status in a group of individuals living below the poverty line in India. They found that individuals with iron deficiency in various age groups (ranging from 5 to 77 years of age) exhibited a significant reduction in transferrin and other salivary protein levels compared to controls [29]. Their findings suggest that nutritional deficiencies can lead to decreased levels of antimicrobial proteins in saliva, potentially compromising oral immunity and increasing the risk of developing dental caries. This aligns with Fonseca’s 2017 review on children’s oral health and malnutrition, which supports Mahadevan’s findings [30].

Furthermore, the presence of ECC can serve as a warning sign for IDA, as illustrated by a case involving a 5-year-old child. This child presented with decayed teeth and tongue sensitivity following the consumption of spicy or hot food. After a routine oral examination, the child was diagnosed with IDA. Peripheral blood smear examination revealed microcytic and hypochromic cells, indicative of ID erythropoiesis, even without the marked Hb level and hematocrit alterations typically associated with anemia. This case underscores that IDA in early childhood may manifest with oral symptoms and highlights the importance of the early diagnosis of systemic conditions presenting with oral manifestations, as it can significantly impact a child’s overall health and well-being [31]. An exfoliative cytology examination of the depapillated areas of the tongue was performed to rule out candidiasis, which presents with similar oral manifestations affecting the tongue, like a burning sensation or tenderness or diffuse or patchy atrophy on dorsal tongue papillae. Oral therapy with FESO4 caused a noticeable rapid initial recovery presenting with a remission of tongue sensitivity and the disappearance of the previous physical findings. This case highlights the fact that prolonged IDA in early childhood may be associated with oral manifestations as well as possible impaired cognitive and motor development. Therefore, the early diagnosis of a systemic condition presenting with oral manifestations can positively impact a child’s health [31,32].

Two investigations have assessed the relationship between IDA and ECC. The outcomes revealed that children with IDA exhibited a higher incidence and severity of ECC. Prolonged breastfeeding up to the age of two was associated with an increased likelihood of both IDA and ECC. Furthermore, a maternal history of IDA during pregnancy was identified as a contributing factor for heightened risk. Moreover, children who did not receive iron supplements exhibited a heightened risk for both ECC and IDA. These findings underscore the intricate interplay of socio-economic determinants, maternal well-being, and the efficacy of supplementation in influencing the occurrence of ECC and IDA, thereby providing valuable insights for both clinical management and public health interventions [33,34] (Table 1).

Animal studies have provided valuable insights into the role of iron in dental caries. These experiments have shown that the administration of iron compounds like FeSO4 or FeCl3 can reduce the incidence of dental caries compared to that in control groups [42]. In studies involving desalivated rats, dietary iron supplementation was found to lower the incidence of smooth-surface carious lesions [43]. Previous research conducted on both animals and humans has also demonstrated that adding iron to cariogenic diets can lead to a reduction in dental caries [44,45]. While some experimental animal studies have directly assessed the impact of ID diets on caries risk, it is worth noting that dietary iron content can be a complex factor to isolate, as cariogenic diets can differ in various aspects [46]. Iron plays a role in giving rat and mouse incisors their characteristic brown pigmentation, and it also contributes to protecting enamel by forming an acid-resistant layer on the surface. This layer acts as a replacement mineral for ions lost during the demineralization process, which is a key aspect of dental caries development [47,48].

Our laboratory aimed to determine whether mice with IDA and infected with *S. mutans* had more severe carious lesions compared to mice fed a normal iron diet. To investigate this, three-week-old C57BL/J6 mice were fed a cariogenic diet containing either standard iron levels (48 ppm Fe) or low iron levels (4 ppm Fe). The results of blood analysis at the end of the fifth week revealed that mice on the ID diet exhibited low Hb and HCT levels, indicating IDA. The IDA mice infected with *S. mutans* had the highest caries severity scores when compared to normal infected mice. Additionally, the mice infected with *S. mutans* and fed a standard iron diet showed lesion numbers and severity scores similar to those of the uninfected IDA mice [49].

A similar study was conducted by Xu et al. wherein rats were inoculated with *S. mutans* and subjected to different iron diets (8 ppm and 45 ppm), followed by an evaluation of caries scores after a 3-month period. Their findings revealed that a low-iron diet exacerbated the pathological damage caused by dental caries in young rats, resulting in more severe enamel demineralization. Furthermore, the IDA group exhibited significantly more extensive dentinal lesions in the sulcal surface compared to the control groups. Importantly, the various iron levels administered to the rats did not have any discernible impact on the morphological structure of their salivary glands. These studies provide valuable new insights into the pathogenesis of dental caries and offer important guidance for clinical prevention strategies. Furthermore, they suggest that iron may play a role in the pathological damage caused by childhood caries by influencing enamel mineralization [50]. Several studies have also explored the impact of iron on oral microbial populations. It has been reported that iron can reduce bacterial biofilm growth and enamel demineralization in a dose-dependent manner in both in vitro and in vivo settings [42,51,52,53]. Iron’s anti-caries effect may be attributed to its ability to inhibit the F-ATPase enzyme of *S. mutans* and influence the acidogenicity and aciduricity of this bacterium [54]. Other researchers have observed a dose–response relationship between iron concentration and reductions in both enamel demineralization and the *S. mutans* population in vitro [55,56]. Collectively, these animal studies shed light on the complex relationship between iron, oral microbial communities, and dental caries, providing valuable insights into the potential mechanisms by which ID may contribute to an increased risk of caries development.

The relationship between iron and its effects on caries virulence factors, as well as the potential mechanisms underlying this relationship, is complex and sometimes conflicting. Some studies have reported that iron can interfere with sucrose metabolism, potentially reducing the production of extracellular polysaccharides (EPSs) and inhibiting the glycosyltransferase (GTF) enzymes produced by *S. mutans* in vitro [57,58]. This inhibition may occur through an oxidative mechanism involving a Fenton-type reaction. However, there are also conflicting observations. Some studies, such as that conducted by Ribeiro and others, did not find an iron-dependent inhibition of EPS production in vitro, in rodent models, or in situ [55,58,59]. These conflicting findings highlight the complexity of the interactions between iron and caries virulence factors, and more research is needed to fully understand these mechanisms.

It is also worth considering that IDA might affect oral defenses rather than solely enhancing the aggression of cariogenic pathogens. For example, dry mouth (xerostomia) has been observed in a significant percentage of adult patients with IDA [60]. Additionally, iron-binding proteins like lactoferrin and transferrin play important roles in iron homeostasis and the immune system. These proteins can bind to planktonic bacteria in saliva and bacteria adherent to teeth or mucosal surfaces. However, transferrin has a weak affinity toward *S. mutans* and can be degraded and utilized by subgingival plaque bacteria [61,62]. Human and animal studies on ID have shown reduced bactericidal activity due to a decreased NADPH-dependent oxidative burst, reduced activity of iron-containing enzymes in neutrophils (myeloperoxidases), and decreased production of interleukin-2 (IL-2) by lymphocytes [63,64]. Understanding the role of iron-binding proteins in IDA and dental infections is essential for unraveling the relationship between ID and caries. In summary, while there is evidence from observational studies and laboratory/animal models suggesting a link between IDA and susceptibility to dental caries, more rigorous epidemiological studies and clinical trials are needed to confirm and further explore the role of IDA correction in caries prevention. The interactions between iron, cariogenic pathogens, and host defenses are complex, and additional research is required to gain a comprehensive understanding of this relationship.

## 4. Iron Deficiency Anemia and Its Impact on Periodontal Diseases

**Figure 3:**
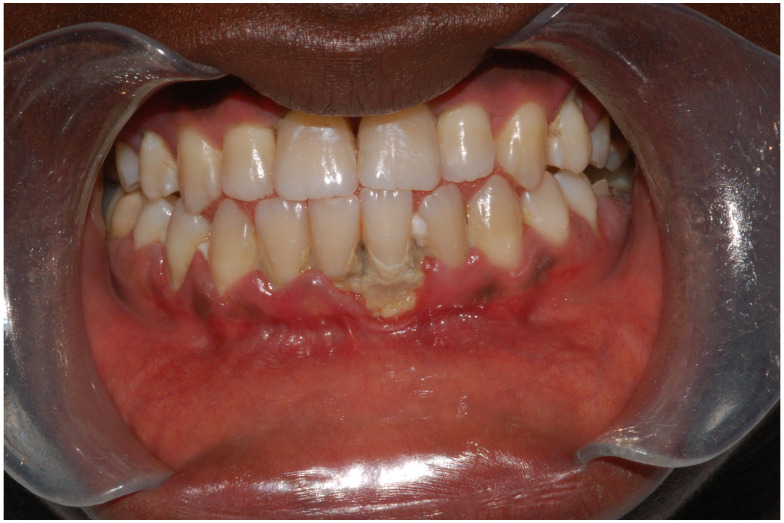
Legends. Intraoral photograph of 13-year-old male showing a punched-out gingival lesion adjacent to the lower central incisors, resulting in a loss of the interdental papilla. Lesions such as these are a consequence of acute necrotizing ulcerative gingivitis (ANUG). This patient was a recent immigrant to the United States from West Africa, a region where iron deficiency is common. The patient’s oral cavity harbored Aggregatibacter actinomycetemcomitans, an organism associated with local aggressive periodontal disease.

The significance of iron in periodontal health and the effects of anemia have been widely researched, with a particular focus on its general impact rather than on iron deficiency anemia (IDA) specifically, which has been less extensively covered. Research reveals a consistent association between chronic periodontitis and lower levels of Hb, HCT, and red blood cell (RBC) counts, highlighting studies spanning from 2009 to 2023 [65,66]. Moreover, data from the US National Health and Nutrition Examination Survey demonstrate a modest, yet statistically significant, increased risk of severe periodontal disease in individuals with insufficient iron intake, suggesting a parallel between the prevalence of IDA, caries, and periodontal diseases within certain populations. A notable case involves a 13-year-old male, a recent immigrant to the US from West Africa, a region prone to ID, who presented with gingival lesions indicative of acute necrotizing ulcerative gingivitis (ANUG). The patient was also infected with Aggregatibacter actinomycetemcomitans, a bacteria associated with aggressive periodontal disease (Figure 3). 

Two studies have delved into the potential biological connection between IDA and periodontal disease, albeit with varied outcomes. Enhos and colleagues examined the periodontal status of IDA patients, focusing on serum and gingival crevicular fluid (GCF) ferritin levels post-periodontal therapy, but found no significant correlation, suggesting IDA’s limited role as a periodontal disease risk factor. Conversely, Chakraborty et al. investigated the activity of the superoxide dismutase (SOD) enzyme in the saliva and serum of chronic periodontitis patients, with and without IDA. The findings indicated that patients with IDA exhibit more severe periodontal symptoms and significantly lower levels of SOD, implying that IDA could exacerbate oxidative stress and contribute to the pathogenesis of chronic periodontitis [67,68].

A recent study by Boychuk-Tovsta et al. (2021) focused on the impact of ID on mineral and trace element levels in the blood and oral fluids of pregnant women with generalized periodontitis. They found that pregnant women with mild to moderate ID had significantly reduced levels of essential minerals and trace elements, including calcium, iron, copper, and zinc, in comparison to a control group of pregnant women without pregnancy complications but with similar stages of periodontitis. This decrease in mineral levels was also observed in the oral fluids, suggesting that both IDA and periodontitis progression can disrupt mineral homeostasis [69]. These studies collectively underscore the intricate relationship between IDA and periodontal disease, implicating factors like inflammation, cytokine production, and iron availability. They call for longitudinal research to further elucidate this connection and highlight the importance of addressing nutritional deficiencies and improving overall health in susceptible populations to mitigate the risks of both IDA and oral diseases (Table 2).

## 5. Oral Manifestations of IDA and Its Association with Oral Candidiasis

ID and its more severe form, IDA, are implicated in various oral health conditions, including tongue pain and oral candidiasis. Candida albicans, a pathogen involved in both oral and systemic diseases, thrives by sequestering iron from the host, employing sophisticated mechanisms for iron acquisition such as high-affinity reductive systems, siderophore uptake, and hemoglobin-iron assimilation [78]. Though ID alone may not solely drive chronic oral Candida infections, it significantly contributes to their persistence. In examining the direct effects of ID on oral health, studies show that prolonged anemia can lead to glossal atrophy, dysphagia, and tongue pain. A specific study assessed eighteen individuals with ID and seven anemic patients who experienced spontaneous tongue pain without visible lesions. Anemic patients not only had a longer history of tongue pain but also exhibited more frequent painful oral sites with lower pain thresholds compared to the ID group. Both stimulated and non-stimulated salivary flow rates were significantly reduced in these groups. Oral iron supplementation in these patients increased serum ferritin levels, enhanced pain thresholds, improved salivation, and reduced tongue pain within two months [70].

Similarly, another study reported that atrophic lingual changes linked to candidiasis were prevalent among forty individuals with tongue pain triggered by spicy or hot foods. Among them, predisposing factors for candidiasis like diabetes, malignancy, or prolonged antibiotic therapy were common. Only a small fraction had mild anemia or ID, with C. albicans identified in 72.5% of cases. Antifungal therapy significantly alleviated or completely resolved tongue pain in 80% of treated patients, along with regeneration of the filiform papillae [71]. Wu et al. (2014) compared oral symptoms and blood characteristics between IDA patients and healthy controls. The IDA group showed a significantly higher incidence of symptoms such as burning oral mucosa, lingual varicosity, xerostomia, oral lichen planus, and atrophic glossitis. They also exhibited lower average levels of hemoglobin, red blood cell count, mean corpuscular volume, iron, and vitamin B12 compared to controls [60]. Another study identified a significant prevalence of oral mucosal changes and Candida infections among patients with ID or IDA, underscoring the diagnostic value of these oral manifestations [72]. Overall, the spectrum of oral conditions associated with ID and IDA is extensive. These conditions include atrophic glossitis, burning mouth syndrome, oral lichen planus, recurrent aphthous stomatitis, Behcet’s disease, oral submucous fibrosis, and oral precancer, demonstrating varying degrees of ID and IDA prevalence [74,77]. These findings highlight the complex interactions between iron levels and oral health, emphasizing the need for comprehensive assessments to diagnose and treat these conditions effectively.

## 6. Oral Manifestations of IDA and the Association of IDA with Oral Ulcerative Diseases

Angular cheilitis is an inflammatory condition marked by erosive inflammation at the mouth’s corners, characterized by redness, flaking, cracking, and ulcers. Contributing factors include nutritional deficiencies, local and systemic causes, and side effects from medications. This condition often affects individuals who wear dentures or mouth masks and young children who use pacifiers, which may lead to drooling [79]. Barnadas et al. (1999) examined the prevalence of deficiencies in iron, folic acid, and vitamin B12 among 80 patients with recurrent oral ulcerations (RUO), comparing these to a control group of 29 patients with various other oral diseases. They found that 26.2% of patients with RUO had nutrient deficiencies: 18 had isolated deficiencies—iron in 4 patients, folic acid in 10, and vitamin B12 in 4. Additionally, 3 patients had combined deficiencies of these nutrients, suggesting that individuals with recurrent oral ulcerations are more likely to have deficiencies in these nutrients compared to those with other oral mucosal diseases [75].

Challacombe et al. (1983) also reported a high prevalence of low ferritin levels among patients with recurrent oral ulceration, specifically 15% in those diagnosed with Behçet’s disease and 9.5% in those with other ulcerative oral conditions, compared to less than 3% in controls [73]. Similarly, Sumathi et al. (2014) found that 66% of patients exhibited reduced ferritin levels compared to healthy controls. These studies highlight the importance of ferritin as an indicator of iron status in individuals with oral mucosal lesions [76]. IDA can compromise cellular immunity, exacerbating the vulnerability to mucosal diseases. Severe clinical manifestations of IDA may include glossitis, dysphagia, and the formation of esophageal webs, symptoms characteristic of Plummer–Vinson syndrome (PVS). This syndrome, predominantly observed in female patients, can be ameliorated by correcting the underlying iron deficiency, potentially reversing the dysphagia [80] (Table 2). Collectively, these findings underscore the critical role of iron in maintaining mucosal health and suggest that managing anemia could mitigate some of the severe complications associated with IDA in the oral cavity and upper digestive tract.

**Significance:** This comprehensive review article delves into the intricate connections between DA and various oral health conditions, specifically oral candidiasis, dental caries, and periodontal diseases. The significance of this exploration lies in its profound implications for both clinical practice and public health. Understanding these intricate relationships can provide healthcare providers with the knowledge required to identify oral manifestations of IDA early, allowing for timely interventions. Furthermore, this review underscores the relevance of addressing nutritional deficiencies, particularly IDA, within vulnerable populations, such as children and those living in poverty. Improved oral health not only enhances the overall well-being of individuals but can also contribute to the betterment of entire communities, highlighting the broader public health relevance of this research.

**Future Directions:** Several promising avenues of research and intervention have emerged from the findings discussed in this review. First and foremost, conducting longitudinal studies to establish definitive cause-and-effect relationships between IDA and oral health conditions is of paramount importance. These studies should encompass diverse populations and account for potential confounding factors to yield more robust insights. Additionally, interventional research can play a pivotal role in examining the impact of iron supplementation or dietary modifications in preventing or managing dental caries and periodontal diseases among individuals with IDA. It is also imperative to delve deeper into the underlying mechanisms that link IDA with oral health, encompassing aspects such as enamel development, oral microbiota, and host immune responses. In terms of public health strategies, tailored interventions are needed to address IDA and its repercussions for oral health, especially within at-risk populations. This may involve initiatives like nutritional education programs, iron supplementation efforts, and projects promoting enhanced access to oral healthcare. Furthermore, exploring the utility of specific biomarkers in saliva or blood with respect to identifying individuals at risk of oral health issues due to IDA can significantly aid in early detection and intervention. The establishment of clear treatment protocols for healthcare professionals dealing with the oral health issues of individuals with IDA is another vital step. Lastly, fostering multidisciplinary collaboration between dentists, pediatricians, hematologists, and nutritionists can facilitate a holistic approach to managing both IDA and oral health. In summary, this review article not only sheds light on the complex interplay between IDA and oral health but also sets the stage for future research endeavors and healthcare strategies aimed at improving health outcomes for affected individuals.

## Figures and Tables

**Table 1:** Influence of IDA on the incidence of dental caries: detailed examination.

IDA and Caries	Observations	Ref.
ECC	ECC can serve as a warning sign for IDA, as illustrated by a case involving a 5-year-old child. This child presented with decayed teeth and tongue sensitivity following the consumption of spicy or hot food.	[31]
	This study revealed a statistically significant correlation between serum iron levels and ECC but not with respect to ferritin. It concluded that there was an inverse relationship between serum iron levels and dmft index scores among preschool children.	[17]
	This case study highlights a link between dental caries and IDA in a 3-year-old boy, whose dental crowns were severely damaged by caries. Laboratory tests revealed decreased hematocrit and low levels of hemoglobin, ferritin, and serum iron. Additionally, the boy exhibited impaired growth and delays in neuropsychomotor development.	[35]
	This cross-sectional study involved 160 preschool children, observing a correlation between their Hb levels and the dmft index scores. The children diagnosed with anemia exhibited significantly higher mean dmft index scores when compared to their non-anemic counterparts.	[14]
	This study investigating the relationship between serum ferritin levels in children under the age of six and the severity of ECC found significantly lower mean ferritin levels in ECC patients compared to a caries-free group.	[20]
	This study assessing the relationship between serum iron and ferritin levels and dental caries in children found a significant association between the dmft index and serum iron, but not ferritin, levels.	[21]
	The study investigated salivary ferritin levels in 120 children, comparing 60 with severe early childhood caries (S-ECC) to a caries-free control group. The results revealed significantly higher salivary ferritin levels in the S-ECC group compared to the caries-free group.	[36]
	The findings revealed that the IDA ECC group exhibited a higher oral flora diversity, whereas the BSCF group showed a lower diversity. Bacterial genera such as Bacillus, Moraxella, and Rhodococcus were significantly present in the IDA ECC group, while Neisseria was more prevalent in the NIDA ECC group.	[37]
	In a cross-sectional study aimed at exploring the association between IDA and ECC, 80 children were enrolled, comprising 40 with diagnosed IDA and 40 healthy controls. The results indicated a significant negative correlation between dmft scores and both Hg and MCH levels, alongside a positive correlation between caries experience and the presence of anemia.	[23]
	This cross-sectional study involving 1598 children revealed several factors positively correlated with caries incidence. These included a history of IDA during pregnancy, preexisting IDA among children below 2 years of age, breastfeeding up to 2 years of age, low socio-economic status, and a lack of iron supplementation.	[34]
S-ECC	In total, 74% of children with S-ECC had blood hemoglobin levels ranging from low to borderline, and 80% had low serum ferritin levels. These findings suggest that IDA may serve as a risk marker for S-ECC.	[16]
	This study evaluated 160 preschool children with S-ECC undergoing full dental rehabilitation. Their findings did not reveal a significant association between Hb or HCT levels and S-ECC. However, they did observe a significantly lower MCV in S-ECC patients.	[15]
	In this study, the objective was to evaluate the levels of ferritin, Hb, and iron in patients both before and 4-6 months following dental stem cell (SC) restoration. The findings revealed that, initially, children exhibited low levels of ferritin and hemoglobin, indicative of IDA.	[26]
	Koppal et al. conducted a cross-sectional study to investigate the relationship between IDA and S-ECC. They concluded that ECC and IDA are certainly correlated, but a longitudinal study is recommended to further investigate the role of IDA as a risk factor for ECC.	[18]
	In this study, 150 children who participated, initial assessments showed that 53% were anemic, 30% had ID, and 20% were dealing with IDA. Follow-up assessments were conducted with 106 children, revealing significant improvements in levels of ferritin and hemoglobin, a 16% decrease in ID cases, and a 20% reduction in IDA cases from baseline to follow-up.	[38]
	Tang et al. examined 101 children to understand the relation between IDA and S-ECC. A multivariable logistic regression analysis found that children with dmfs that are higher or equal to 35 have a 7.25-fold higher risk of developing IDA.	[19]
	In a 2016 study by Bansal et al., 60 children aged 2–6 were assessed to examine the link between severe early childhood caries (S-ECC) and iron deficiency anemia (IDA). The study compared 30 children with S-ECC to 30 caries-free controls and found a significantly higher prevalence of IDA in the S-ECC group, and lower levels of Hb, MCV, and PCV in children with S-ECC.	[22]
	This study assessing children with S-ECC undergoing full dental rehabilitation under general anesthesia revealed no correlation between Hb and HCT levels and S-ECC. However, S-ECC patients exhibited a significantly lower MCV.	[15]
	This study aimed to assess whether children with S-ECC are more likely to have anemia and lower levels of vitamin D than those without S-ECC, alongside examining factors tied to elevated parathyroid hormone (PTH) levels, previously linked to S-ECC. The research included 266 children, 144 of whom had S-ECC, with an average age of about 41 months. The findings revealed that those with S-ECC were significantly more prone to having deficiencies in both vitamin D and hemoglobin, exhibited higher levels of PTH, and were more likely to suffer from iron-deficiency anemia compared to the control group.	[39]
	The study encompassed 266 children, with 54.14% suffering from severe caries (SC) and 45.86% being caries-free. Logistic regression analysis revealed that children with SC were nearly twice as likely to exhibit reduced ferritin levels and had a six-fold higher risk of this condition compared to the caries-free group.	[40]
	The study demonstrated that children with S-ECC had significantly lower levels of hemoglobin and ferritin. They were over six times more likely to have iron deficiency anemia (IDA) compared to their peers without dental caries. Additionally, the frequency of IDA was significantly higher in S-ECC children compared to that in the caries-free control group.	[10]
	The study explored potential associations between S-ECC and serum iron and ferritin levels in children. Out of a total of 688 children, 82 were chosen based on their decayed, missing, and filled primary teeth (dmft) scores. The findings revealed statistically significant differences solely in the dmft and unsaturated iron-binding capacity values, with no significant differences observed in any other blood parameters.	[25]
	This study involving 204 child–parent pairs showed that anemia-related ECC negatively affected both the child and their parents’ quality of life. It impacted the child’s function, image, and psychology while also causing distress for the parents.	[32]
	This study aimed to evaluate and compare anthropometric measurements, hemoglobin levels, and salivary parameters between healthy children and those with S-ECC aged 3–6 years. The findings revealed significant differences across all anthropometric measurements. Notably, children suffering from severe dental caries exhibited low hemoglobin levels, a condition that, if persistent, could result in anemia.	[41]

**Table 2:** Impact of iron deficiency anemia on periodontal and other oral diseases: a comparative analysis.

IDA and Oral Diseases	Observations	Ref.
Periodontal diseases	After periodontal therapy, the periodontal status of IDA patients revealed no correlation between changes in serum and GCF ferritin levels. This suggests that IDA is not a direct risk factor for periodontal diseases.	[68]
	IDA-CP patients displayed elevated gingival index values, bleeding on probing, probing pocket depth, and percentages (%) of sites with clinical attachment loss compared to CP patients. Moreover, the mean salivary and serum SOD levels were notably lower in IDA-PH, CP, and IDA-CP patients compared to the control group (CG).	[67]
	This study explored the effects of ID on mineral levels in pregnant women with generalized periodontitis. The study compared 42 affected women to 34 without pregnancy complications, finding significant decreases in serum calcium, iron, copper, and zinc levels in the affected group. These reductions suggest that iron deficiency and periodontitis disrupt the mineral balance in biological fluids.	[69]
Candidiasis and other oral manifestations	A similar study also explored the link between ID and glossal pain, comparing 18 patients with ID to 7 with anemia but no visible oral abnormalities. It found that anemic patients experienced more prolonged and widespread tongue pain.	[70]
	This study investigated the link between atrophic tongue changes and candidiasis in 40 patients experiencing tongue pain with spicy or hot foods. In the study, 60% of the participants had predisposing factors for candidiasis, such as diabetes, malignancy, or long-term antibiotic use. Blood tests showed mild anemia or iron deficiency in 12.5% and mild diabetes in 10%, with the majority (72.5%) showing normal levels. C. albicans was detected in 72.5% of cases through culture tests.	[71]
	IDA patients had significantly higher frequencies of all oral manifestations than healthy controls; a burning sensation of oral mucosa, lingual varicosity, dry mouth, oral lichen planus, and atrophic glossitis were the five leading oral manifestations for IDA patients.	[60]
	ID predisposed patients to a high incidence of Candida infection and showed a variety of oral manifestations including angular cheilitis, atrophic glossitis, pseudomembranous candidosis, erythematous candidosis, median rhomboid glossitis, chronic mucocutaneous candidosis, papillary hyperplastic candidosis, and cheilocandidosis.	[72]
	Patients with recurrent oral ulceration exhibited low ferritin levels, with a specific emphasis on certain conditions. For example, 15% of individuals with Behcet’s disease and 9.5% with other ulcerative oral lesions were found to have low ferritin levels, in stark contrast to the less than 3% prevalence observed among control subjects.	[73]
	The analysis shows a varying prevalence of a specific deficiency across different oral conditions: 16.9% in atrophic glossitis, 20.3% in burning mouth syndrome, 13.6% in oral lichen planus, 20.1% in recurrent aphthous stomatitis, 34.9% in Behcet’s disease, 20.6% in oral submucous fibrosis, and 21.4% in oral precancer.	[74]
Ulceration	The purpose of this study was to determine the prevalence of iron, folic acid, and vitamin B12 deficiencies among 80 patients with recurrent oral ulcerations (RUO) when compared to a control group consisting of 29 patients with various other oral diseases. In the group of patients with recurrent oral ulcers, deficiencies were found in 21 out of 80 patients. Among these cases, 18 had isolated deficiencies: iron in 4 patients, folic acid in 10 patients, and vitamin B12 in 4 patients. In addition, 3 patients had combined deficiencies of these nutrients.	[75]
	Significantly decreased ferritin values were observed in ulceration patients compared to healthy subjects.	[76]
	The prevalence of IDA varies among ulcerative conditions, with 2.8% of atrophic glossitis patients, 7.9% of those with recurrent aphthous stomatitis, 6.3% of Behcet’s disease patients, and 1.5% of oral submucous fibrosis patients diagnosed with IDA.	[77]
